# Suppression of corticospinal excitability by sleep spindles without increase in GABAergic inhibition

**DOI:** 10.64898/2026.09.03.748984

**Published:** 2026-09-08

**Authors:** Friederike Breuer, Sianna Groesser, Katrin Himmerich, Katrin Knietsch, Umair Hassan, Saman Seifpour, Anastasiia Grigoreva, Ulf Ziemann, Til Ole Bergmann

## Abstract

Thalamocortical sleep spindles are hypothesised to support memory consolidation during sleep by creating transient windows of enhanced hippocampal-neocortical communication and synaptic plasticity. A recent real-time electroencephalography (EEG)-triggered transcranial magnetic stimulation (TMS) study found a pulsed suppression of corticospinal excitability during spindles relative to spindle-free non-rapid eye movement (NREM) sleep, driven by the spindle falling phase. We hypothesised that this phasic suppression may reflect local inhibitory network dynamics, measurable as GABA-A receptor-mediated short-interval intracortical inhibition (SICI) using paired-pulse TMS. We applied real-time EEG-triggered single- and paired-pulse TMS over the primary motor cortex during pre-sleep wakefulness, spindle-free N2/N3 sleep, and at four sleep spindle phases (peak, falling, trough, and rising). Corticospinal excitability was strongly reduced from wakefulness to spindle-free N2/N3 sleep, and further suppressed during sleep spindles. Numerically, excitability was lowest during the falling phase and trough, although we found no significant modulation across spindle phases. Contrary to our hypothesis, neither spindle presence nor phase significantly modulated SICI. Secondary analyses provided preliminary evidence that slow oscillations present at stimulation increased excitability and reduced SICI, irrespective of spindle presence. Together, these findings indicate distinct contributions of sleep/wake vigilance states, sleep spindles, and slow oscillations to cortical network dynamics, and provide new insight into the transient modulation of corticospinal excitability and GABA-A-receptor mediated SICI during human NREM sleep.

## Full Text Availability

The license terms selected by the author(s) for this preprint version do not permit archiving in PMC. The full text is available from the preprint server.

